# A Lightweight convolutional neural network for nicotine prediction in tobacco by near-infrared spectroscopy

**DOI:** 10.3389/fpls.2023.1138693

**Published:** 2023-05-12

**Authors:** Di Wang, Fengyuan Zhao, Rui Wang, Junwei Guo, Cihai Zhang, Huimin Liu, Yongsheng Wang, Guohao Zong, Le Zhao, Weihua Feng

**Affiliations:** ^1^ Zhengzhou Tobacco Research Institute of China National Tobacco Corporation (CNTC), Zhengzhou, China; ^2^ School of Mathematics and Statistics, Zhengzhou University, Zhengzhou, China; ^3^ Technology Center of China Tobacco Guizhou Industrial Co., Ltd., Guiyang, China

**Keywords:** tobacco, near-infrared spectroscopy, nicotine, lightweight, convolutional neural network

## Abstract

The content of nicotine, a critical component of tobacco, significantly influences the quality of tobacco leaves. Near-infrared (NIR) spectroscopy is a widely used technique for rapid, non-destructive, and environmentally friendly analysis of nicotine levels in tobacco. In this paper, we propose a novel regression model, Lightweight one-dimensional convolutional neural network (1D-CNN), for predicting nicotine content in tobacco leaves using one-dimensional (1D) NIR spectral data and a deep learning approach with convolutional neural network (CNN). This study employed Savitzky–Golay (SG) smoothing to preprocess NIR spectra and randomly generate representative training and test datasets. Batch normalization was used in network regularization to reduce overfitting and improve the generalization performance of the Lightweight 1D-CNN model under a limited training dataset. The network structure of this CNN model consists of four convolutional layers to extract high-level features from the input data. The output of these layers is then fed into a fully connected layer, which uses a linear activation function to output the predicted numerical value of nicotine. After the comparison of the performance of multiple regression models, including support vector regression (SVR), partial least squares regression (PLSR), 1D-CNN, and Lightweight 1D-CNN, under the preprocessing method of SG smoothing, we found that the Lightweight 1D-CNN regression model with batch normalization achieved root mean square error (RMSE) of 0.14, coefficient of determination (*R* ^2^) of 0.95, and residual prediction deviation (RPD) of 5.09. These results demonstrate that the Lightweight 1D-CNN model is objective and robust and outperforms existing methods in terms of accuracy, which has the potential to significantly improve quality control processes in the tobacco industry by accurately and rapidly analyzing the nicotine content.

## 1 Introduction

The tobacco industry occupies a key position in the economic development of China (Zhao, 2022). The research found that the quality of tobacco leaves directly affects the quality of cigarette products. Nicotine, an alkaloid found in the Solanaceae family, is one of the important components of tobacco leaves. Especially, the nicotine content of key chemical components plays an important role in assessing the quality of tobacco leaves in general (Henry et al., 2019). Furthermore, besides harm to brain activity and respiratory health, long-term exposure to nicotine can do even more damage to the body and negatively affect concentration and memory. Thus, rapid measurement and stable regulation of nicotine content are especially critical for industrial companies to produce tobacco products that meet industry requirements. However, lab-grade conventional measurement methods for nicotine generally include several complex processes, such as grinding samples with special characteristics, preparations of extraction reagent, and calculations using experimental data (Hossain and Salehuddin, 2013), which are always time-consuming, contaminated, and laborious. As a result, selecting a rapid, cost-effective, and robust analytical technique to assess nicotine content in tobacco leaves is paramount.

**Figure 1:**
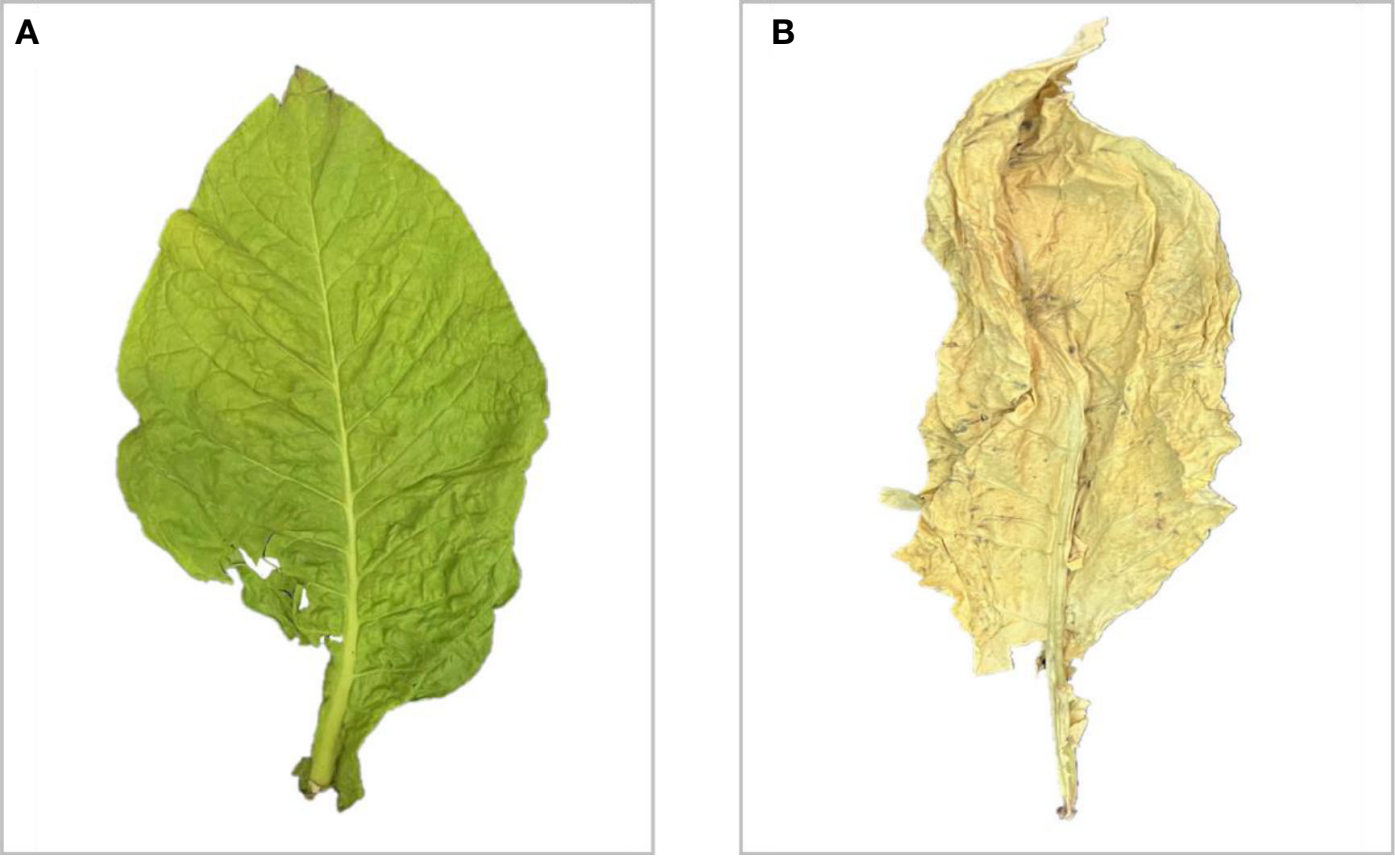
The appearance of tobacco leaves in different periods. **(A)** Fresh. **(B)** After the process of roasting.

Because chemical substances of the tobacco leaves are complex, nicotine is the most important alkaloid in tobacco, which directly affects the quality and industrial availability of tobacco leaves. The appearances of tobacco leaves in different periods are shown in **Figure 1**. **Figure 1A** illustrates the appearance of fresh tobacco leaves during the growth of the tobacco plant. The growth and development of tobacco plants are affected by various environmental factors including ecology, soil, and fertilizer, which can alter the synthesis and accumulation of substances such as nicotine. **Figure 1B** displays the tobacco leaves after they have undergone the process of roasting. Determining the nicotine content of tobacco leaves after roasting is a necessary step in the industrial production process to ensure that only leaves that meet the necessary requirements are selected for use. The chemical constituents of tobacco plants can be significantly affected by soil improvement and fertilization practices. In this paper, the research mainly focuses on the rapid detection and quantitative analysis of nicotine based on near-infrared (NIR) spectroscopy. The ability to determine nicotine content rapidly at different stages of plant growth can provide valuable information for the targeted control of nicotine content through the manipulation of external factors such as soil fertilization and moisture levels. Integrating computer science and plant science can help optimize tobacco production and improve the quality of tobacco leaves field (Huang and Shu, 2021).

In recent years, NIR spectroscopy as the representative one of non-destructive analysis technologies has been widely used in the tobacco industry, which can be employed to measure the quality and safety attributes of tobacco leaves and tobacco products (Bi et al., 2019). As a natural plant, tobacco leaves contain a large number of functional groups such as C–H, O–H, N–H, and C═O, which are often analyzed by NIR spectroscopy to reduce the environmental pollution caused by reagents used and time consumption in conventional chemical analysis (Roy et al., 1993). Previous studies have shown that the chemometrics models built using the partial least squares (PLS) method for the quantitative analysis have been used to predict the chemical composition of the target in agricultural products, for example, sugar, protein, and alkaloid content (McGlone and Kawano, 1998; Blanco and Peguero, 2010). Additionally, studies that focused on the classification problem, near-infrared spectra combined with pattern recognition techniques, divide the sample into several categorical class labels (Kim et al., 2000). Rapid determination of chemical composition in tobacco leaves by NIR has become one of the hot spots in tobacco chemistry research.

Generally, the regression methods can be used to model the relationship between the NIR spectroscopy and the concentration of the analyte. However, multicollinearity always occurs when predictor variables are highly correlated to each other in the regression model. That makes it difficult to specify the model’s interpretability and meets an overfitting problem (Wondola et al., 2020). Previous studies have reported numerous successful applications of machine learning methods, such as support vector regression (SVR) (Leng et al., 2021), backpropagation (BP) neural networks (Li et al., 2021), and artificial neural network (ANN) (Mutlu et al., 2011) on spectral data, which have shown many advantages when compared to the partial least squares method normally used for solving such high multicollinearity problems. Meanwhile, the number of NIR spectral wavelengths is so much more than representative samples, which will decrease the generalized performance of the quantitative analysis model. Hence, there is a growing body of research in the spectral pre-processing methods for wavelength selection and dimensionality reduction, including the successive projection algorithm (SPA) (Liu et al., 2015), principal component analysis (PCA) (Toscano et al., 2017), and wavelet transform (WT) (Li et al., 2020). Unfortunately, the above algorithms will require scientists to master even more mathematical skills and knowledge to obtain multiple necessary parameters, which will reduce the efficiency of model construction. The past decade has seen the great development of artificial intelligence. Convolutional neural network (CNN) is the most representative technique of deep learning, which is one of the most commonly used in the data analysis (Alzubaidi et al., 2021). Numerous studies have shown much successful application with CNN, especially in the field of computer vision (Yang et al., 2020), natural language processing (Young et al., 2018), speech recognition (Adel-Hamid et al., 2014), etc. In the research of tobacco NIR spectroscopy, several studies used a deep convolutional neural network algorithm to classify the regions of tobacco leaves (Wang et al., 2020). In other studies, a fully convolutional network has been developed in the quantitative analysis domain of tobacco leaves to construct analytical models and predict the nicotine volume of tobacco leaves (Jiang et al., 2021). It has been demonstrated that deep learning methods can extract useful features automatically from high-dimensional NIR spectral data without any feature selection methods.

We summarized the existing methods for predicting targets using NIR spectroscopy in **Table 1**. Multiple linear regression uses multiple explanatory variables for quantitative analysis (Liu et al., 2015), and noise in one or more of the independent variables can have a substantial impact on the accuracy of the model, which is prone to noise and has a slow rate of convergence. Kernel regression methods are sensitive to outliers, which can be computationally expensive, especially for large datasets (Mora and Schimleck, 2009). Artificial neural network regression models are prone to overfitting without any regularization, which is not acceptable in spectral analysis (Mutlu et al., 2011). An improved deep CNN classification model was proposed to recognize and discriminate tobacco cultivation regions accurately. However, the pooling operation used in this model may reduce its overall representational capacity (Wang et al., 2020). Selecting the optimal number of Synergy PLS regression model factors and wavelength intervals can be time-consuming (Sampaio et al., 2018). The regression based on iterative PLS is extremely sensitive to the initial values of parameters and more computationally intensive than other regression techniques in the quantitative analysis of NIR spectra. Therefore, optimizing the parameters requires the expertise of a professional and experienced researcher (Genisheva et al., 2018). MCUVE-PSO-SVR is a combination of multiple techniques, finding that a near-optimal set of hyperparameters can require domain expertise, which is more complex to implement and less interpretable (Wang et al., 2022). While a one-dimensional fully convolutional network (1D-FCN) has been proposed to quantitatively analyze the nicotine composition of tobacco leaves, it did not take into account the impact of noise on the dataset. As a result, the accuracy of the predictive nicotine content may be affected (Jiang et al., 2021). Based on the prior research, an imperative in model construction involves the implementation of facile data pre-processing techniques, a parsimonious network architecture, a diminished count of layers, and a thorough evaluation of overfitting propensity.

**Table 1:** Comparison table of related work.

Proposed method	Preprocess method	Limitations
Consensus successive projection algorithm (SPA)–multiple linear regression (MLR) (Liu et al., 2015)	SG	Sensitive to noise and slow to converge
Kernel regression methods (Mora and Schimleck, 2009)	SNV, MSC, SG	Sensitive to outliers and computationally expensive
Artificial neural network regression model (Mutlu et al., 2011)	None	Prone to overfitting without any regularization
Improved deep CNN classification model (Wang et al., 2020)	None	Pooling can reduce the representational capacity of model
Synergy PLS regression model (Sampaio et al., 2018)	SNV, MSC, SG	The number of PLS factors and wavelength interval selection are time-consuming
New iterative PLS regression model (Genisheva et al., 2018)	SNV	Sensitive to the initial values of the parameters and more computationally expensive
MCUVE-PSO-SVR model (Wang et al., 2022)	SNV, SG	More complex to implement and less interpretable
A 1D-FCN model (Jiang et al., 2021)	None	Does not consider the impact of noise on the dataset

SG, Savitzky–Golay; SNV, standard normal variate; MSC, multiplicative scatter correction; CNN, convolutional neural network; PLS, partial least squares.

Hence, the main purpose of this study is to develop a new approach for predicting the nicotine content of tobacco leaves. This paper proposed a CNN‐based method for quantitative modeling of the NIR spectral dataset, composed of multiple building blocks, such as four convolution layers and one fully connected layer. In the meantime, a technique called batch normalization was used to make the training of neural networks faster and more stable between convolution layers (Ioffe and Szegedy, 2015), choosing the rectified linear unit (ReLU) activation function for each deep learning network to reduce the likelihood of vanishing gradients (Agarap, 2018).

## 2 Materials and methods

### 2.1 Data collection

#### 2.1.1 Near-infrared spectral dataset

In this study, all samples of tobacco leaves were collected from provincial tobacco industrial companies in China, which were the most representative sample in 2020. Relying on reference standards in the tobacco industry, the sample can be crushed with a finger after the drying process. The Retsch Ultra Centrifugal Mill ZM 200 is used to grind materials to produce particles with a diameter of 1–10 μm. The resulting powder is sieved through a 0.250-mm (60 mesh) sieve, and the particles that pass through the sieve are mixed and placed in a sealed bag.

**Figure 2:**
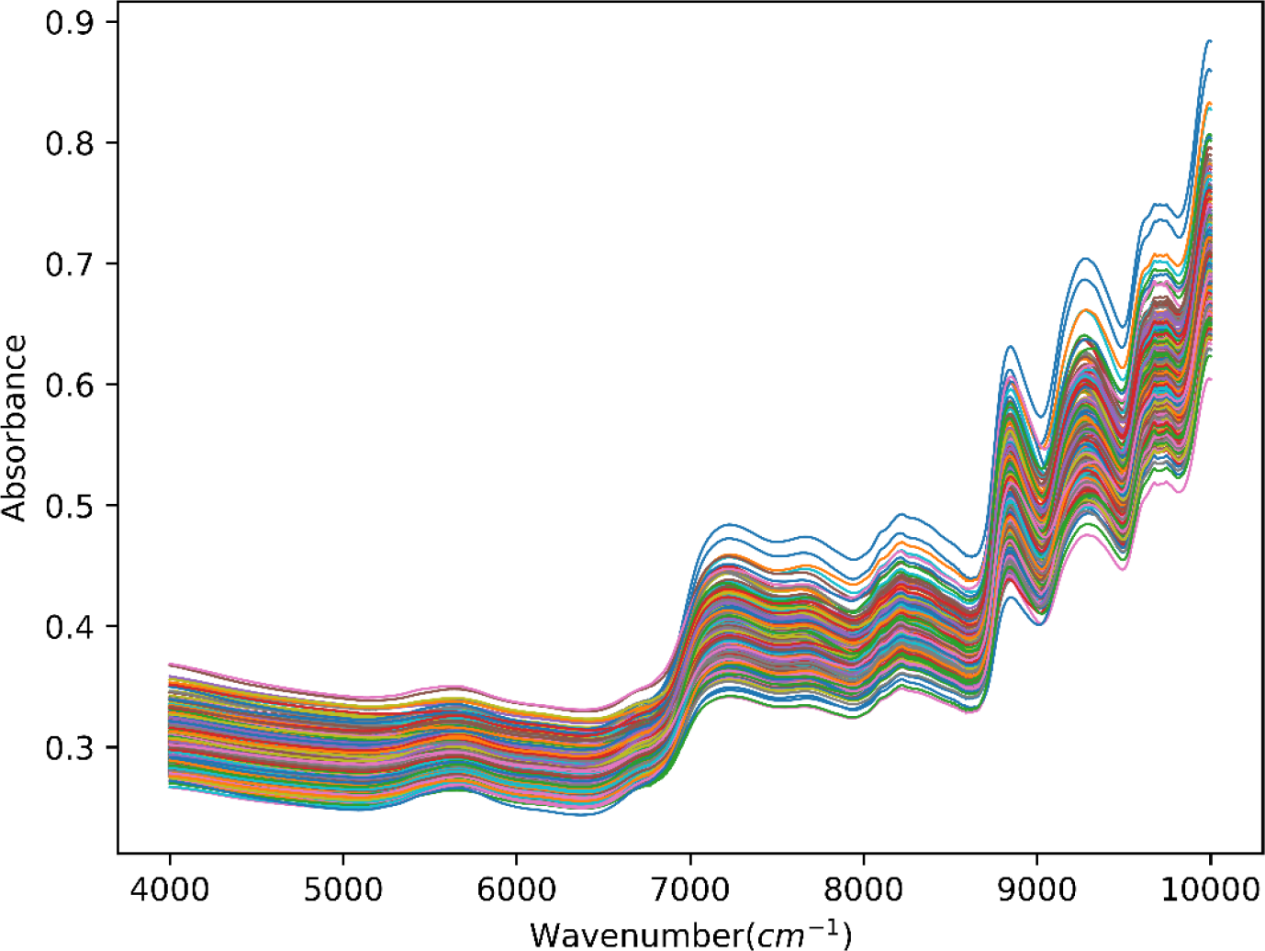
NIR spectra for tobacco leaves collected from various samples. NIR, near-infrared.

Spectra of tobacco leaves were acquired with the analytical instrument, which is the MPA II FT-NIR spectrometer made by Bruker. We have set a series parameter of equipment, such as the resolution is 8 cm^−1^, the number of scans is 64, and the NIR spectral region is from 3,999 to 10,001 cm^−1^. As a result, the averaged NIR spectra of 620 samples were collected for analysis, which is shown in **Figure 2**. The appearance and trend of spectral profiles of different samples exhibited similar shapes and trends. Still, some variations in absorbance reflected the different accumulation of chemical components in different samples.

#### 2.1.2 Nicotine content acquisition

As an instrumental analysis method, the continuous flow technique has been generally used for rapid and accurate analysis of samples. After a series of sample preparation procedures, tobacco leaves’ nicotine content was measured with the analytical standards in the tobacco industry.

### 2.2 Preprocessing of spectral data

In the original data space, one of the first crucial steps in deep learning (DL) is the preprocessing of the dataset. Through the comprehensive analysis of the spectral data, some problems were found during the process of modeling, such as being inconsistent, erroneous, and missing. Based on the literature review and the research finding, there are several existing preprocessing techniques to assure model robustness.

To minimize the unwanted negative influence of any external factors on the measured spectra, especially the effects of instrument noise, the standard normal variate (SNV) algorithm calculates the mean and standard deviation over the spectral wavelengths of all samples. Its purpose is to reduce the potential impact of different measurements (Mishra and Lohumi, 2021). In the multiplicative scatter correction (MSC) method (Helland et al., 1995), it is assumed that scattering effects in spectral data cause a shift in the baseline of the spectra. The average spectrum is calculated and used as a reference spectrum to correct this shift. However, NIR spectral signals are highly overlapping and strongly correlated; SNV is exclusively used to eliminate the effect on the spectra due to the uneven distribution of particle, without considering the other random noise. Moreover, it is supposed that spectral data follow a normal distribution, and the dataset may lose some necessary information for model calibration after SNV processing. By contrast, the Savitzky–Golay (SG) filter has been developed as a popular method for spectral smoothing. (Savitzky and Golay, 1964), which can locally fit a specialized polynomial of moving window to remove an amount of noise and improve the signal-to-noise ratio (SNR) of a spectral dataset. The desired signal of the original dataset was a significant enhancement, as well as the excellent and much more efficient preservation of sharp absorbance peak.

**Figure 3:**
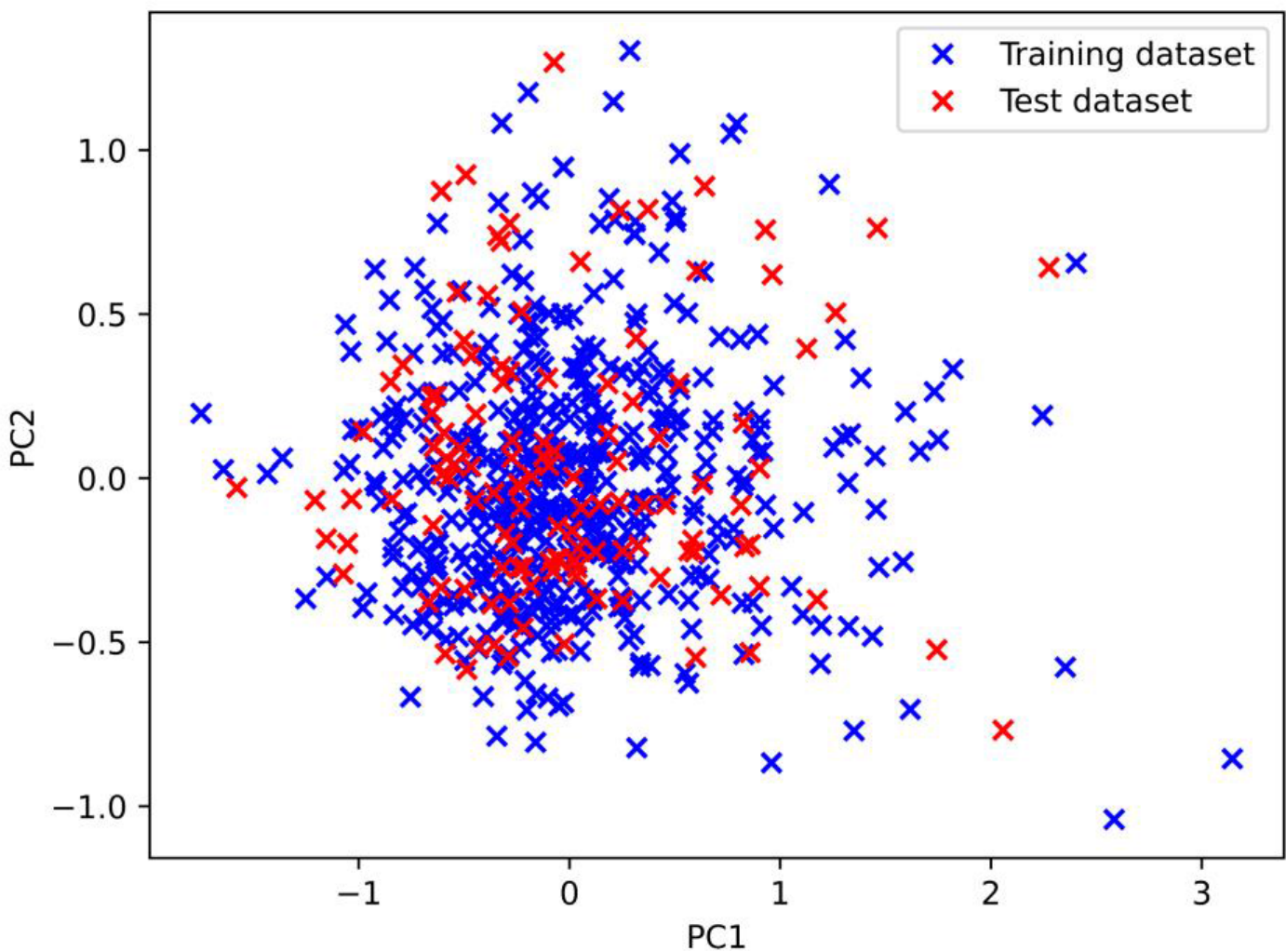
The distribution of the training dataset and test dataset under the first two principal components.

**Figure 4:**
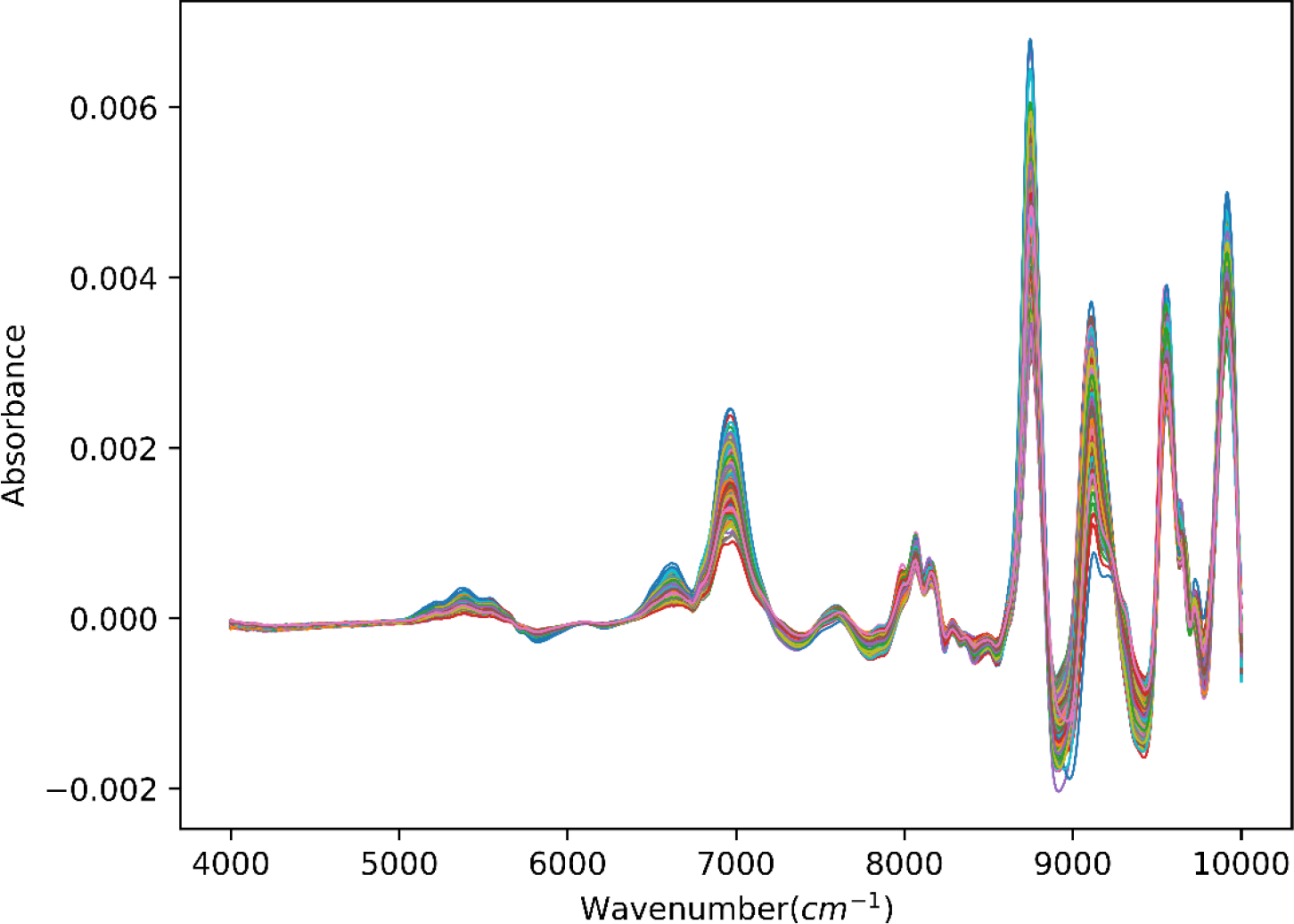
NIR spectra for tobacco leaves after SG smoothing preprocess. NIR, near-infrared; SG, Savitzky–Golay.

In this research, all spectral data with obvious errors are eliminated. To avoid an overfitting problem, we assume the spectral dataset was divided into two pieces by the train_test_split function from the scikit-learn module (Bi and Hu, 2020): 80% is the training dataset (validation set is taken out of them), and the remaining 20% is the test dataset. After the application of PCA to the overall dataset, it was observed from **Figure 3** that the distribution of the divided training dataset and the test dataset was consistent under the first two principal components. This indicates that the split strategy is suitable for use in machine learning models, as the training and test datasets are representative of the same underlying distribution. At the same time, SG smoothing is selected for preprocessing; the results are displayed in **Figure 4**. Previous parameter optimization of experiments shows that the following set of parameter values gave the best results (Rozov, 2020): window_length = 17, polyorder = 2, and deriv = 1.

### 2.3 Lightweight 1D-CNN model

A CNN-based prediction model for nicotine content is proposed in this paper. The goal of CNN is to discover the relationship between the input feature and the target data (O'Shea and Nash, 2015). The input is convolved with multiple convolutional layers, enabling the network to obtain and implicitly weigh the contributions of the unintuitive feature. In order to minimize the estimated prediction error, the backpropagation algorithm is applied during the training process. This algorithm updates the model’s parameters by calculating the gradient of the loss function with respect to the input data and the model’s parameters. The Lightweight 1D-CNN model consists of multiple convolutional layers that extract features from the one-dimensional spectral signal of tobacco leaf samples. This model is designed to accelerate the prediction of tobacco nicotine content.

**Figure 5:**
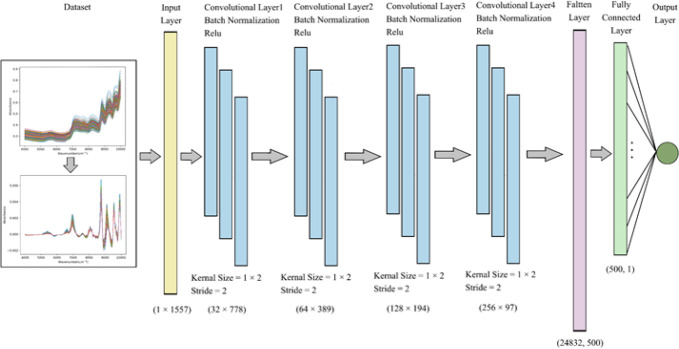
Schematic diagram of Lightweight 1D-CNN model. 1D-CNN, one-dimensional convolutional neural network.

The present information in **Figure 5** shows that the Lightweight 1D-CNN architecture consists of seven layers: one input layer, four convolutional layers, one flatten layer, one fully connected layer, and one output layer. After the data preprocessing, all training datasets through the input layer are calculated by convolutional layers, which effectively extract the features of the high-dimensional spectral dataset with a set of filters. In general, the purpose of pooling layers is to reduce the dimensions of the feature map and increase the receptive field (Tolias et al., 2015). Considering the limited number of spectral samples, this work does not add the pooling operation to the back of individual convolutional layers. As a linear transformation on the input vector, each neuron is connected to the output node by a weight matrix within the dense layer. In addition, instead of a random dropout operation, batch normalization is selected in CNN to keep the spectral variance relatively stable, speed up model training, and enhance the generalization capability (Li et al., 2019). For each layer, the ReLU is set as an activation function to overcome the problem of vanishing or exploding gradients, which can enhance the weight sparsity of the network. Finally, models are optimized in this research by minimizing mean squared error (MSE). The nicotine content is generated from the prediction model based on CNN and the original input spectra.

As can be seen from **Table 2**, for each sample, the NIR spectra have 1,557 points, which are taken as one-dimensional spectral input features. Each convolutional layer has 32, 64,128, and 256 channels with a kernel of size 1 × 2 and a stride of 2, separately. After the fourth convolution operation, the data size is reshaped from 256 × 97 to 1 × 24,832, which represents the input vector to a fully connected layer. In quantitative analysis of nicotine, as a regression issue, the result is computed by the fully connected layer and the output node.

**Table 2:** The architecture settings of Lightweight 1D-CNN model.

Layer	Name	Channels	Size
1	Inputs	1	(1, 1,557)
2	Covolution1 Batch normalization ReLU	32 - -	(32, 778) - -
3	Covolution2 Batch normalization ReLU	64 - -	(64, 389) - -
4	Covolution3 Batch normalization ReLU	128 - -	(128, 194) - -
5	Covolution4 Batch normalization ReLU	256 - -	(256, 97) - -
6	Flatten layer	–	24,832
7	Fully connected ReLU	- -	500 -
8	Output	1	1

1D-CNN, one-dimensional convolutional neural network; ReLU, rectified linear unit.

Specifically, we choose several hyperparameters that control the performance of the model, such as the optimizer function, learning rate, batch size, weight decay, and the number of epochs. The values of these hyperparameters are shown in **Table 3**. Instead of the classical gradient descent procedure, the Adam optimizer can be used to update network weights iteratively based on the spectral training dataset (Kingma and Ba, 2014). The learning rate is one of the most important hyperparameters, which controls how much to adjust the model weights in response to the estimated error each time. The batch size determines the number of training samples that will be processed through the model at once; all the samples in the same batch size will be trained together as a group. Weight decay is the most widely used regularization technique during training to improve generalization performance by reducing complexity. The number of epochs defines the number of times the complete training dataset will be propagated through the neural network, which equals the number of iterations if the entire training dataset is the batch size. We can improve the model’s performance and accuracy by carefully choosing these hyperparameters.

**Table 3:** The training hyperparameters settings of CNN-based model.

Parameter	Settings	
Optimizer	Adam
Learning rate	1 × 10^−4^
Batch size	16
Weight decay	1 × 10^−8^
Epochs	1,000

CNN, convolutional neural network.

For an enhanced demonstration of the superiority of our proposed model, we have conducted an analysis by comparing two distinct forms of neural network models (1D-FCN (Jiang et al., 2021) and Lightweight 1D-CNN) with regard to their parameters count, number of layers, and average prediction time, and the results are presented in **Table 4**. The Lightweight 1D-CNN model is more space-efficient and faster in terms of prediction time, while the 1D-FCN model is more complex.

**Table 4:** The regression report of the proposed model.

Parameter	1D-FCN	Lightweight 1D-CNN
Number of model parameters	591,901,201	12,504,521
Number of layers	9	7
Average time for prediction (min)	204	32

1D-FCN, one-dimensional fully convolutional network; 1D-CNN, one-dimensional convolutional neural network.

In the current study, we design a predictive model using Python and the PyTorch framework (1.10.2). The CNN network architecture was implemented using PyTorch. The model was trained and evaluated on a workstation equipped with an i9-12900K CPU, 256G RAM, and two NVIDIA 24GB GeForce RTX 3090 GPUs. The runtime environment for the python program was created using Docker and Windows subsystem for Linux (WSL2), which allowed for efficient and stable execution of the program.

### 2.4 Evaluation metrics

In order to evaluate the generalization performance of the proposed model, there are three popular metrics selected for analysis: root mean square error (RMSE), coefficient of determination (*R* ^2^), and residual prediction deviation (RPD). Meanwhile, we compared the performance of different regression models between partial least squares regression (PLSR), SVR, 1D-CNN, and Lightweight 1D-CNN approaches to explore the best quantitative analysis method.

The RMSE is a measure of the difference between the measured values and the predicted values of a regression model. It is commonly used to evaluate the average performance over the whole dataset, which can be calculated by Equation 1, where the variable n denotes the number of samples in the test dataset, and ${\hat{y}}_{i}$ and $y_{i}$ are the predicted value and the measured value of the i th test sample, respectively.


$$RMSE=\sqrt{\frac{\sum_{i=1}^{n}{\left({\hat{y}}_{i}-y_{i}\right)}^{2}}{n}}.\;\;\;\;\;\;\;\;\;\;\;\;\;\;\;\;\;\;\;\;\;\;\;\left(1\right)$$

The *R* ^2^ is an important measure of the fit and accuracy of different models in NIR spectroscopy, which can be calculated by Equation 2, where SSE denotes the residual sum of squares, SST is the total sum of squares, and $\bar{y}$ is the mean measured value of the test dataset. The value of *R* ^2^ lies between 0 and 1, with higher values indicating a stronger and more accurate model.


$$R^{2}=1-\frac{SSE}{SST}=1-\frac{\sum_{i=1}^{n}{\left({\hat{y}}_{i}-y_{i}\right)}^{2}}{\sum_{i=1}^{n}{\left(y_{i}-\bar{y}\right)}^{2}}.\;\;\;\;\;\;\;\;\;\;\;\;\;\;\;\;\;\left(2\right)$$

The RPD is the ratio of the standard deviation (SD) of the measured value to the RMSE, which can be calculated by Equation 3. The findings from prior studies classified RPD as follows (Viscarra Rossel et al., 2006): RPD less than 1.0 indicates a very poor predictive model, a value between 1.0 and 1.4 indicates poor model predictions, a value between 1.4 and 1.8 indicates fair model, a value between 1.8 and 2.0 indicates good model, a value between 2.0 and 2.5 indicates very good model predictions, and a value higher than 2.5 indicates excellent model.


$$RPD=\frac{SD}{RMSE}=\frac{\sqrt{\frac{1}{n}\sum_{i=1}^{n}{\left(y_{i}-\bar{y}\right)}^{2}}}{\sqrt{\frac{1}{n}\sum_{i=1}^{n}{\left({\hat{y}}_{i}-y_{i}\right)}^{2}}}.\;\;\;\;\;\;\;\;\;\;\;\;\;\;\;\;\;\;\left(3\right)$$

## 3 Results

### 3.1 The preprocessing method

**Figure 6:**
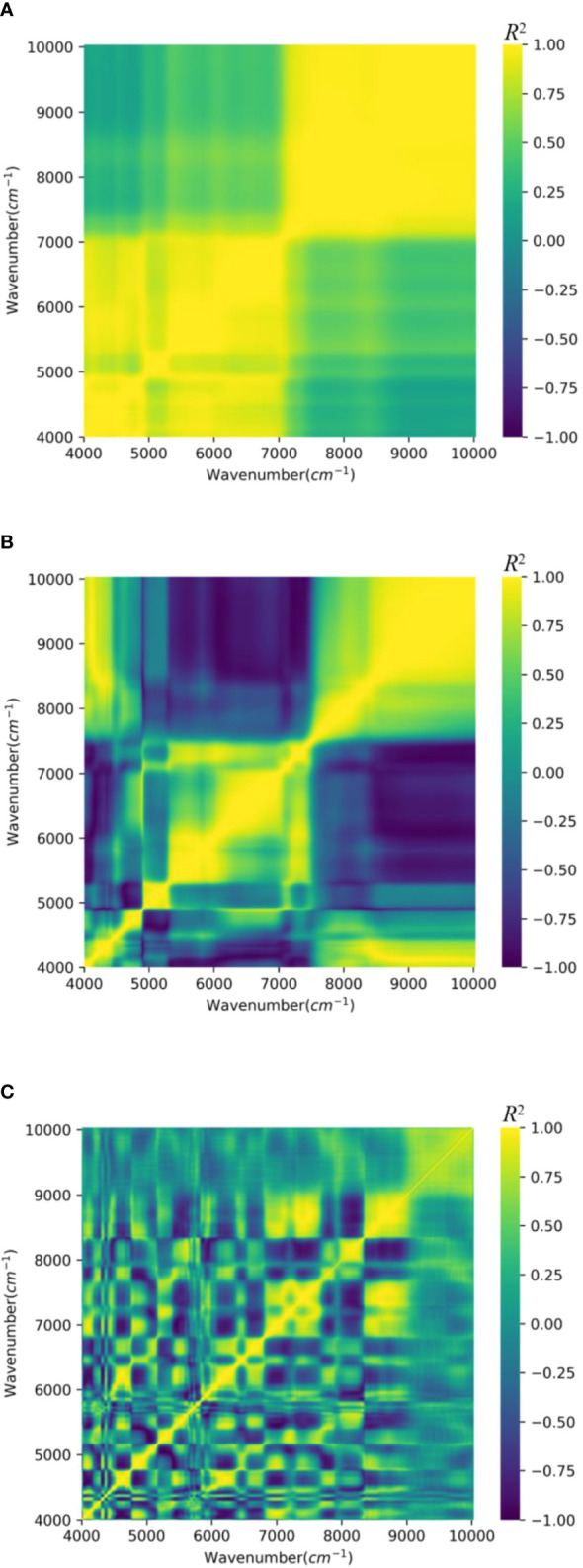
Wavelength–wavelength two-dimensional correlation of NIR spectra. **(A)** Raw data. **(B)** MSC. **(C)** SG. NIR, near-infrared; MSC, multiplicative scatter correction; SG, Savitzky–Golay.

Numerous studies have found that the NIR spectral signals have a great deal of information: the amount of substance and configuration of a molecule, even though there are some problems such as sample thickness, the noise of the instrument, and baseline drift, which will cause great trouble in the qualitative and quantitative analysis of samples. In this research, the collected samples of tobacco leaves were preprocessed by SG smoothing. The comparison before and after spectral preprocessing, as shown in **Figure 6**, indicates that the original spectra were greatly affected by noise signal and that the multicollinearity problem among the wavenumbers is serious. In particular, **Figure 6A** shows that the predictor variables in the wavenumber ranges of 4,000–7,000 and 7,000–10,000 (the region of yellow values) are more strongly correlated with each other; their correlation coefficients (r) are between 0.75 and 1. **Figure 6B** presents the correlation between the predictor variables after applying the MSC method. Compared to the original data, the wavenumber bands below 7,500 showed a reduction in multicollinearity; other wavenumber bands did not show a significant improvement. **Figure 6C** is the correlation heatmap of spectra after SG smoothing, and the predictor variables in a total of approximately 10 wavenumber bands (4,000–4,100, 4,500–4,700, 4,700–5,000, 5,200–5,500, 6,200–6,500, 6,500–6,800, 7,000–7,700, 8,000–8,200, 8,200–9,000, and 9,000–10,000) are strongly correlated with one another.

The RMSE results of the prediction model with different preprocessing methods are shown in **Table 5**. The Lightweight 1D-CNN achieved the same RMSE of 0.04 when applied separately to the raw data and the data preprocessed using the MSC and SG methods on the training dataset. When the model was tested on the independent test dataset, the RMSE values were 0.24, 0.14, and 0.21 for the raw data, the MSC-processed data, and the SG-processed data, respectively. Moreover, it is evident that other models outperformed both the MSC-processed data and the raw data when applied to the SG-processed data. The SG method could be selected for data preprocessing during the model construction to improve model performance.

**Table 5:** The RMSE results of models with different preprocessing methods.

Preprocessing method	Raw		SG		MSC	
	Training	Test	Training	Test	Training	Test
1D-CNN	0.29	0.31	0.24	0.29	0.25	0.32
Lightweight 1D-CNN	0.04	0.24	0.04	0.14	0.04	0.21
PLSR (Gowen et al., 2011)	0.22	0.45	0.14	0.31	0.05	0.42
SVR (Leng et al., 2021)	0.24	0.33	0.25	0.29	0.59	0.63

RMSE, root mean square error; SG, Savitzky–Golay; MSC, multiplicative scatter correction; 1D-CNN, one-dimensional convolutional neural network; PLSR, partial least squares regression; SVR, support vector regression.

### 3.2 Performance analysis of different models

In this paper, we aimed to reduce the complexity of neural networks by using regularization techniques, such as dropout and batch normalization, during training. We also minimize the impact of data preprocessing on model construction by using cross-validation to evaluate the performance of different models on a held-out test dataset.

**Figure 7:**
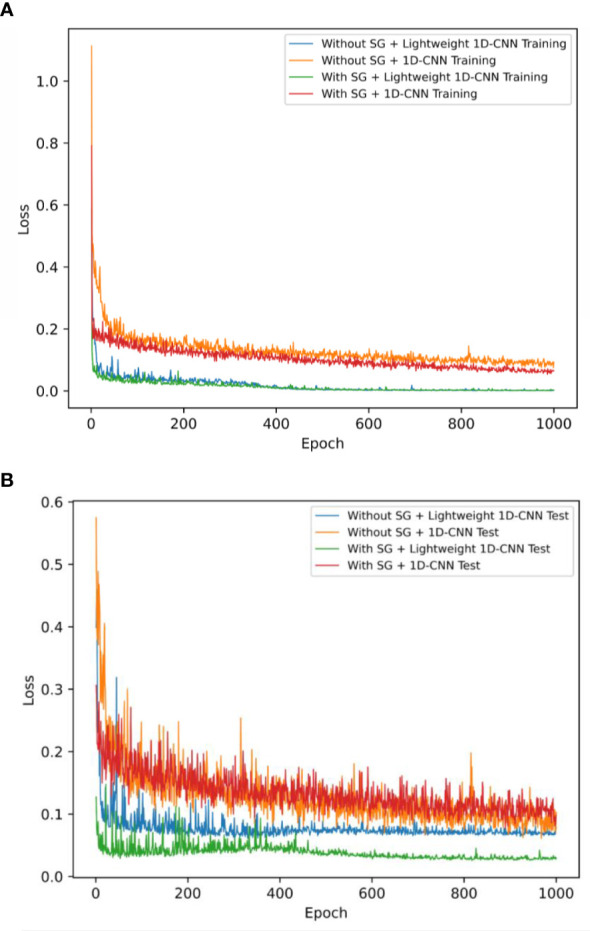
The mean squared error loss of Lightweight 1D-CNN and 1D-CNN models with and without SG smoothing on two different datasets. **(A)** Training dataset. **(B)** Test dataset. 1D-CNN, one-dimensional convolutional neural network; SG, Savitzky–Golay.

To clearly demonstrate the effects of SG smoothing and dropout on model performance, we established four different CNN models to predict the nicotine content of tobacco leaves. After odd spectral samples were excluded, we split the remaining 617 samples into a training dataset of 493 samples and a test dataset of 124 samples using an 80/20 strategy. The MSE loss during the training and test process is shown in **Figure 7**. As can be seen from **Figure 7A**, for the CNN models without SG smoothing, the training loss values are both relatively large initially. Especially 1D-CNN, using the dropout technique, has a larger initial loss value, which is higher than 1.1, and the minimum loss value is 0.15 at the end of training. At the same time, 1D-CNN and Lightweight 1D-CNN are both trained with 1,000 epochs after SG smoothing; their loss values drop really fast to 0.02, and it has been observed that the Lightweight 1D-CNN model without the dropout procedure converges faster than the other one. From **Figure 7B**, we can see that after preprocessing the data with SG smoothing, we finished the training process, and the loss function of the Lightweight 1D-CNN has the minimum value on the test dataset, which is 0.05. In another model with dropout regularization, 1D-CNN reaches convergence with the same preprocessing method, and its loss value is 0.15. The remaining two models, without any preprocessing applied, have the same performance in terms of loss values, which are 0.1 and 0.12, respectively.

In order to compare the generalization performance of different models after SG smoothing, we evaluated the CNN models with various structures and traditional chemometrics methods like PLSR and SVR. The experimental results of the training dataset and test dataset are shown in **Table 6**. The experimental results showed that the Lightweight 1D-CNN model performed better on the regression problem without making use of dropout compared with PLSR and SVR. The model has an RMSE of 0.04 and an *R* ^2^ of 0.99 on the training dataset and an RMSE of 0.14 and an *R* ^2^ of 0.95 on the test dataset. In contrast, the 1D-CNN model does not perform well enough, as evidenced by its low *R* ^2^ of 0.79 on the test dataset and 0.83 on the training dataset. As shown, PLSR performed better than SVR at predicting the content of nicotine on the training dataset, but the model performed poorly on the test dataset. Regarding RPD, we only consider the model’s performance on the test dataset. The proposed Lightweight 1D-CNN model has the largest RPD of 5.09 among the models tested, as shown in **Table 6**. This suggests that the Lightweight 1D-CNN model is more effective at achieving the desired outcome compared to the other models.

**Table 6:** The performance results of 1D-CNN, Lightweight 1D-CNN, PLSR, and SVR.

Performance	RMSE		*R* ^2^		RPD
	Training	Test	Training	Test	Test
1D-CNN	0.24	0.29	0.83	0.79	2.76
Lightweight 1D-CNN	0.04	0.14	0.99	0.95	5.09
PLSR (Gowen et al., 2011)	0.14	0.31	0.96	0.78	2.13
SVR (Leng et al., 2021)	0.25	0.29	0.89	0.86	2.67

1D-CNN, one-dimensional convolutional neural network; PLSR, partial least squares regression; SVR, support vector regression; RMSE, root mean square error; RPD, residual prediction deviation.

## Discussion

Confronted with a limited amount of data but a high dimensional NIR spectral dataset, researchers must carefully select and apply appropriate methods to extract meaningful information from the data. Numerous studies have been conducted to investigate the effects of different preprocessing methods on the accuracy and reliability of NIR spectral data.

In the traditional chemometrics analysis, the most commonly used preprocessing methods are SG smoothing, SNV, and MSC, which are applied to correct for various sources of noise and error in the NIR spectral data. The results of the model prediction error regarding the use of different data preprocessing methods are shown in **Table 5**. The results indicate that SG smoothing is more suitable than MSC for the construction of a 1D-CNN model. There are two reasons for this. First, MSC is a parametric method, which means that it makes assumptions about the underlying distribution or shape of the data. Specifically, MSC assumes that the spectral data contain a baseline shift and scatter that can be corrected by dividing the data by a reference spectrum (Smith et al., 2019). On the contrary, as a non-parametric method, SG smoothing does not make any assumptions about the underlying distribution or shape of the data. This makes SG smoothing applicable to a wider range of datasets, regardless of the characteristics of the spectral data (Zhang et al., 2020). Second, MSC is known to sometimes overcorrect the data, particularly when the range of concentrations is large and the SNR is low, which can result in poor prediction accuracy. In contrast, SG smoothing has the advantage of being a non-parametric method, which makes it applicable to a wider range of datasets. The use of SG smoothing has been shown to improve the SNR of the data in several studies and enhance the correlation between wavenumbers in spectral data, which can improve the generalization performance of a prediction model (Acharya et al., 2016). As shown in **Figure 6**, SG smoothing can help make the absorbance peak more distinct by reducing the noise in the data, while preserving the shape and features of the data, which can improve the accuracy and reliability of the analysis. In this study, we explored the use of different methods for data pre-processing when modeling with 1D-CNN, Lightweight 1D-CNN, PLSR, and SVR. As shown in **Table 5**, after comparing the performance of MSC-processed SG-processed and raw data, we found that using SG smoothing provided the best results.

After application of the smoothing technique known as SG preprocessing, the correlation of the data is reduced, as shown in **Figure 6**, which displays the smoothed dataset. We performed a modeling analysis on the smoothed data using several different regression methods, including 1D-CNN, Lightweight-CNN, PLSR, and SVR. As **Table 6** shows, the Lightweight 1D-CNN model had the lowest prediction loss and higher *R* ^2^ and RPD, followed by the SVR, 1D-CNN, and PLSR models. Compared to CNN, SVR is not effective at modeling complex non-linear relationships in the NIR spectral data. Especially in kernel-based SVR, the kernel function is used to transform the input NIR spectral data into a higher-dimensional space. This is necessary because SVR uses a linear decision boundary in the transformed feature space, and the kernel function allows for more complex decision boundaries to be learned. However, SVR is sensitive to the choice of the kernel function, as demonstrated in several studies (Chen et al., 2007; Mora and Schimleck, 2009). When the wrong kernel function was selected, the performance of SVR was consistently poorer than when the appropriate kernel function was used. In addition, PLSR has been demonstrated to be a popular and effective method for modeling NIR spectroscopic data in the literature (Gowen et al., 2011). Nonetheless, PLSR is sensitive to multicollinearity among the predictor variables like wavenumbers in NIR spectral data. If a robust model is to be constructed, finding the best principal components and latent variables is necessary. This process can require a significant amount of time and effort, as it involves carefully selecting the appropriate number of components and optimizing the model using various techniques. According to **Table 6**, the PLSR model is still prone to overfitting despite the use of cross-validation, as indicated by the difference in performance between the training and test datasets. This suggests that PLSR may be particularly susceptible to overfitting in this dataset, and further research is needed to identify potential solutions. In our experiments, the results in **Table 6** show that using batch normalization as a regularization technique in network Lightweight the performance of our model compared to using dropout in the traditional CNN model in terms of RMSE, *R* ^2^, and RPD. The RPD of the Lightweight 1D-CNN model is greater than 2.5, which means that the model has a relatively high degree of accuracy. Batch normalization regularizes the activations of the layers, which has several benefits, including reducing internal covariate shifts and improving the stability of the model’s learning process. In contrast, dropout regularizes only the weights of a model by randomly dropping neurons during training. This can slow down the training process and shift the variance of individual neurons when the model transitions from training to test. Overall, the results provide evidence that using a CNN model with batch normalization can be an effective approach for predicting nicotine concentration by NIR spectra. Further research could investigate the interpretability of the neural network model.

## Conclusion

In this study, based on NIR spectral data, we developed and evaluated a Lightweight 1D-CNN regression model to rapidly and accurately quantify nicotine content in tobacco leaves. The model was trained using a dataset of NIR spectra and corresponding nicotine levels and evaluated using a separate test dataset. The results of experiments indicate that the proposed model achieved higher accuracy and robustness when compared to traditional methods such as PLSR and SVR. Additionally, we implemented SG smoothing as a preprocessing step and added batch normalization to each convolutional layer in place of the dropout used in traditional 1D-CNN models; the performance of the Lightweight 1D-CNN model was further improved, resulting in a statistically significant reduction in RMSE and increases in *R* ^2^ and RPD, when the model was evaluated on the test dataset. Our findings have potential applications in the tobacco industry and may enable more efficient and effective quality control processes. Further empirical studies are required to explore its potential for other applications and enhance the interpretability of the model. In addition, as part of future work, we plan to observe how the amount of training data can affect the deep learning models with data augmentation or generative adversarial network (GAN)-based approaches.

## Data availability statement

The raw data supporting the conclusions of this article will be made available by the authors, without undue reservation.

## Author contributions

WF, LZ: funding acquisition. DW, FZ: methodology and validation. JG, HL, CZ: materials collection, data curation. DW: writing—original draft. LZ, RW: writing—review and editing. YW, GZ: software and supervision. All authors contributed to the article and approved the submitted version.
